# Another Fatality Due to Postpartum Group A Streptococcal Endometritis in the Modern Era

**DOI:** 10.7759/cureus.4618

**Published:** 2019-05-08

**Authors:** Akhil Singhal, Mohammad Alomari, Shivangi Gupta, Shaden Almomani, Shrouq Khazaaleh

**Affiliations:** 1 Internal Medicine, Cleveland Clinic Foundation, Cleveland, USA; 2 Internal Medicine, Cleveland Clinic Foundation, Johnson city, USA; 3 Internal Medicine, Shadan Institute of Medical Sciences, Hyderabad, IND; 4 Internal Medicine, Jordanian Royal Medical Services, Amman, JOR

**Keywords:** toxic shock syndrome, postpartum endometritis, group a streptococcus, non-steroidal anti-inflammatory drugs (nsaid)

## Abstract

Group A streptococcus (GAS) is a rare yet potentially lethal cause of postpartum endometritis. Atypical early presentation and the routine use of post-delivery analgesics which might mask the symptoms preclude timely diagnosis and appropriate management. The invasive disease usually follows a rapidly progressive course that has considerable morbidity and mortality. Streptococcal toxic shock syndrome (TSS) can complicate this condition leading to refractory septic shock and possible death. We hereby present a case of a 42-year-old female patient who developed GAS postpartum endometritis complicated by streptococcal TSS resulting in death despite enormous resuscitative efforts. We aim to increase awareness of this lethal condition highlighting the importance of early recognition and prompt management.

## Introduction

Postpartum endometritis refers to infection of the decidua; it has been notoriously linked to significant postpartum morbidity and mortality. Typically, postpartum endometritis is polymicrobial with group A streptococcus (GAS) being a rare cause. While GAS disease can be non-invasive, more aggressive presentations like toxic shock syndrome (TSS) and necrotizing fasciitis might have a significant disease burden. Even though the incidence of GAS is only limited to 200 cases per year in the United States, as of 2002, the risk of acquiring invasive GAS is 20-fold higher among postpartum women than in non-pregnant women [1].

Delay in the diagnosis of invasive GAS can have catastrophic consequences as mortality approaches 30%-50% [2] once shock occurs. The diagnosis could be challenging with the rather non-specific presenting symptoms and the rarity of the disease. Keeping in mind the high morbidity and mortality of this condition, increased awareness and high index of suspicion are crucial for timely diagnosis and prompt management.

## Case presentation

An otherwise healthy 42-year-old female patient presented to our institution with worsening nausea, vomiting, and abdominal pain of four days’ duration. Her surgical history was significant for an uneventful cesarean section two week prior to presentation. Notably, she was prescribed over-the-counter ibuprofen for postoperative analgesia on discharge and had been taking it consistently. She denied any foul vaginal discharge or bleeding other than the expected lochia.

**Figure 1:**
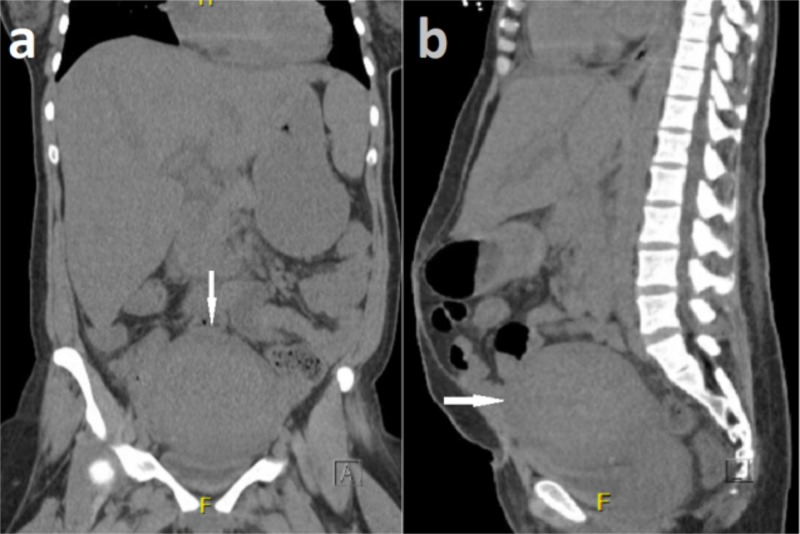
Coronal and sagittal plane computed tomography (a and b, respectively) of the abdomen and pelvis (with contrast) showing bulky uterus with no localized collection of fluid (white arrows)

Upon arrival to the emergency department, her vital signs were significant for tachycardia (heart rate 130 b/m), tachypnea (respiratory rate 31 b/m), and hypotension (blood pressure 77/52 mmHg). Physical examination was consistent with a diffusely tender abdomen, mild rebound tenderness, and trace blood in the vaginal vault. No cervical motion tenderness or purulent material was found on pelvic examination. Laboratory investigations revealed serum lactate of 4.7 mmol/L (normal range: 0.4 - 2.0 mmol/L), white blood count 10.97 k/ul (normal range: 3.70 - 11.00 k/uL), creatinine 3.28 (normal range: 0.70 - 1.40 mg/dL), and bilirubin 2.5 (normal range: 0.0 - 1.5 mg/dL). She had an emergent abdomen and pelvis computed tomography (CT) without contrast which was remarkable for an enlarged postpartum uterus with no localized collection of fluid or other significant abnormalities (Figures 1A-1B).

The patient was resuscitated with 4.5 liters of normal saline; nonetheless, she remained hypotensive, eventually requiring initiation of norepinephrine and transfer to the medical intensive care unit (ICU). She was empirically started on vancomycin, zosyn, and clindamycin for septic shock management. However, the patient remained hypotensive necessitating addition of three more vasopressors, namely norepinephrine, phenylephrine, and epinephrine as well as a stress-dose steroid in efforts to maintain a mean arterial pressure around 60 mmHg. Subsequently, emergent intubation for airways protection was indicated, and just prior to intubation, the patient developed a pulseless electrical activity (PEA) arrest. Cardiopulmonary resuscitation was started with the return of spontaneous circulation achieved after seven cycles. Bedside echocardiogram was done showing an ejection fraction of 30% and severely hypokinetic left ventricle. Surgical and obstetric teams evaluated the patient, and the decision was made to proceed with exploratory laparotomy. The uterus was found slightly enlarged, mottled, and friable requiring hysterectomy and bilateral salpingectomy. Unfortunately, the patient went into another PEA arrest and expired despite all resuscitative measures. Blood culture and surgical pathology results later returned positive for GAS and suppurative endometritis with gram-positive cocci producing chains suggestive of GAS respectively. Autopsy disclosed findings consistent with disseminated intravascular coagulation, diffuse capillary leak, and anasarca supporting the diagnosis of TSS.

## Discussion

According to the World Health Organization, uterine puerperal sepsis is defined as infection that occurs between rupture of membranes and the first 42 days postpartum, with at least two of the following conditions: pelvic pain, fever (defined as oral temperature equal to or higher than 38.5 °C) and purulent, cloudy, or fetid vaginal discharge or delayed uterine involution [3]. The most common risk factors for puerperal infection include cesarean section, prolonged rupture of membranes, extended labor, and vaginal infection before delivery, prior chorioamnionitis, and repeated vaginal examinations with the single most important risk factor being the cesarean section [4].

Incidence of postpartum endometritis is mainly dependent on modes of delivery. The risk with vaginal deliveries is reported to be 1%-3%; however, for scheduled cesarean deliveries it is believed to be 5%-15% and 15%-20% for unscheduled cesarean deliveries [5]. Puerperal infections are usually polymicrobial. The etiologic agents include Chlamydia trachomatis, Neisseria gonorrhoeae, and less frequently Mycoplasma hominis and Mycoplasma genitalium. Other bacteria include Enterobacteriaceae and gram-positive cocci such as Streptococcus species and Enterococcus faecalis, among others [5].

GAS infections in the postpartum period have an estimated mortality rate of 3.5% [1]. The majority of cases present within a week of delivery and generally have a fulminant course [6]. Late-onset cases, though rare, have also been reported [1]. Because postpartum GAS infections remain so uncommon, physicians often misinterpret the pain of developing an infection as usual postpartum discomfort. The widespread use of pain-relieving agents after childbirth can further mask the signs of the incubating infection. Such factors can contribute to a delay in diagnosis, and potential catastrophic outcomes [7]. Our patient's presentation was atypical since her symptoms started relatively late (10 days post-delivery) and she did not develop a fever. Nevertheless, we believe her symptoms could have been masked by the heavy use of non-steroidal anti-inflammatory drugs (NSAIDs).

Streptococcal TSS is an invasive streptococcal infection caused by toxins released by Streptococcus pyogenes, also known as GAS. Multiple streptococcal exotoxins act as superantigens to stimulate T cell activation eventually leading to watershed induction of both T cell lymphocytes and monocytes [8]. A breach of the mucosal barrier during labor and the postpartum period can result in the entry of streptococci into deeper tissues, and eventually the bloodstream, which might lead to streptococcal TSS [9].

Due to the high fatality with GAS infection, it is vital to establish an early diagnosis and start aggressive management. Symptoms could be non-specific ranging from early postpartum fever, chills, nausea, vomiting, and abdominal pain to headaches and delerium [6]. Foul-smelling vaginal discharge may be a cue for diagnosis, but most cases present with scanty to non-purulent odorless lochia [6]. Some of these signs and symptoms may often be masked by the use of pain killers after delivery [10]. Blood and urine cultures should be obtained to confirm GAS. If the uterus is suspected to be the source of infection, endometrial aspiration for a gram stain and culture should be performed. Imaging modalities, such as ultrasound, magnetic resonance imaging (MRI), or CT will typically demonstrate an edematous uterus larger than expected; but it is virtually impossible to distinguish an early GAS infection of the uterine cavity from a normal postpartum uterus, and normal imaging should not delay therapy [11].

Prognosis depends on the timing of treatment and the clinical severity of the illness. As soon as sepsis is suspected, the patient should be started on broad-spectrum antibiotics along with supportive management as indicated. Once GAS infection is confirmed, antibiotics should be escalated to high-dose penicillin combined with clindamycin [9]. Clindamycin is used due to its ability to suppress the synthesis of penicillin-binding proteins and bacterial exotoxins. Also, it has been shown to inhibit bacterial toxin synthesis and promote phagocytosis [12]. Source control and urgent intervention such as surgical exploration, debridement, and rarely hysterectomy are of paramount importance and should not be overlooked in cases with refractory shock [9]. The successful use of intravenous immunoglobulin for exotoxin neutralization in treating streptococcal TSS remains debatable [13].

## Conclusions

Despite the low incidence of GAS endometritis and TSS in pregnant women, this patient population is at a significantly increased risk of complications compared to non-pregnant women. Initial signs of GAS can be easily missed primarily due to the atypical presentation, use of analgesics, and lack of disease awareness which preclude early diagnosis and appropriate management. Therefore, a high index of suspicion is crucial for early recognition of this potentially fatal disease as aggressive medical and surgical interventions can help improve outcomes. This case report is an excellent example of how postpartum GAS with a late presentation and atypical symptoms can quickly become catastrophic despite enormous resuscitative efforts.
